# Public Perceptions and Discussions of the US Food and Drug Administration's JUUL Ban Policy on Twitter: Observational Study

**DOI:** 10.2196/51327

**Published:** 2024-07-11

**Authors:** Pinxin Liu, Xubin Lou, Zidian Xie, Ce Shang, Dongmei Li

**Affiliations:** 1 Department of Computer Science University of Rochester Rochester, NY United States; 2 Goergen Institute for Data Science University of Rochester Rochester, NY United States; 3 Department of Clinical and Translational Research University of Rochester Medical Center Rochester, NY United States; 4 Center for Tobacco Research The Ohio State University Wexner Medical Center Columbus, OH United States

**Keywords:** e-cigarettes, JUUL, Twitter, deep learning, FDA, Food and Drug Administration, vape, vaping, smoking, social media, regulation

## Abstract

**Background:**

On June 23, 2022, the US Food and Drug Administration announced a JUUL ban policy, to ban all vaping and electronic cigarette products sold by Juul Labs.

**Objective:**

This study aims to understand public perceptions and discussions of this policy using Twitter (subsequently rebranded as X) data.

**Methods:**

Using the Twitter streaming application programming interface, 17,007 tweets potentially related to the JUUL ban policy were collected between June 22, 2022, and July 25, 2022. Based on 2600 hand-coded tweets, a deep learning model (RoBERTa) was trained to classify all tweets into propolicy, antipolicy, neutral, and irrelevant categories. A deep learning model (M3 model) was used to estimate basic demographics (such as age and gender) of Twitter users. Furthermore, major topics were identified using latent Dirichlet allocation modeling. A logistic regression model was used to examine the association of different Twitter users with their attitudes toward the policy.

**Results:**

Among 10,480 tweets related to the JUUL ban policy, there were similar proportions of propolicy and antipolicy tweets (n=2777, 26.5% vs n=2666, 25.44%). Major propolicy topics included “JUUL causes youth addition,” “market surge of JUUL,” and “health effects of JUUL.” In contrast, major antipolicy topics included “cigarette should be banned instead of JUUL,” “against the irrational policy,” and “emotional catharsis.” Twitter users older than 29 years were more likely to be propolicy (have a positive attitude toward the JUUL ban policy) than those younger than 29 years.

**Conclusions:**

Our study showed that the public showed different responses to the JUUL ban policy, which varies depending on the demographic characteristics of Twitter users. Our findings could provide valuable information to the Food and Drug Administration for future electronic cigarette and other tobacco product regulations.

## Introduction

Electronic cigarettes (e-cigarettes) have rapidly gained popularity in recent years. These battery-powered devices heat nicotine, flavorings, propylene glycol or vegetable glycerin, and other additives to produce a vapor that users inhale [1,2]. The use of e-cigarettes has been on the rise in recent years, especially among youth and young adults. According to the Centers for Disease Control and Prevention National Youth Tobacco Survey in 2021, among students who currently used each respective tobacco product, frequent use (on ≥20 days of the past 30 days) was 39.4% for e-cigarettes, making them the most commonly used tobacco product among this group [3]. The survey found that 11.3% of high school and 2% of middle school students reported using e-cigarettes [3]. In 2022, 14.1% of high school students and 3.3% of middle school students reported e-cigarette use [4]. These statistics indicate a worrying trend of increasing e-cigarette use among youth in the United States. One e-cigarette brand that has dominated the US e-cigarette market is the JUUL e-cigarette system, which accounts for 75% of the market share in 2018 [5]. The compact design, high nicotine levels, and wide range of flavors of JUUL have made it a popular e-cigarette product choice among teenagers. In 2019, JUUL was the most commonly used e-cigarette brand among US high school students, with more than 59% of high school e-cigarette users reporting current use [6].

e-Cigarette use is associated with respiratory disorders, mental health issues, cognitive impairment, and cancer [7-10]. Respiratory symptoms were more likely to be comentioned with several JUUL flavors (such as mango and mint) by Reddit users [11]. Moreover, the high nicotine levels in JUUL and other e-cigarettes can increase the risk of addiction to other substances, which is especially concerning for young adults [12]. The alarming trend of increasing e-cigarette use among youth and young adults calls for effective measures to regulate the sale and marketing of e-cigarettes and to raise awareness about their potentially harmful effects.

On January 2, 2020, the US Food and Drug Administration (FDA) released the e-cigarette flavor enforcement policy to prohibit the sale of all flavored cartridge-based e-cigarettes, except for menthol and tobacco flavors. Twitter (subsequently rebranded as X) users’ perceptions of e-cigarettes became more negative after the announcement [13]. Furthermore, the FDA has implemented several policies to restrict youth access to flavored e-cigarettes including requiring age verification for web-based sales and restricting the sale of flavored e-cigarettes to age-restricted physical stores [14]. On June 23, 2022, the FDA banned JUUL products from being sold in the United States by issuing marketing denial orders (MDOs) [15]. However, the agency has since put an administrative hold on the ban until it can review JUUL’s marketing application again. On July 5, 2022, the FDA administratively stayed the MDO, as it determined that scientific issues unique to the JUUL application warrant additional review [15]. This administrative stay temporarily suspends the MDO during the additional review but does not rescind it.

Twitter is a popular social media platform with over 200 million active daily users as of March 2023. Twitter data have been used to examine public perceptions and discussions of regulatory policies on tobacco products [13,16,17]. This study aims to provide important insights into public perceptions of the JUUL ban policy and the differences between Twitter users with different demographics. It could inform future e-cigarette regulations and health education campaigns to reduce the harms of e-cigarette use. This study will contribute to the current literature on e-cigarette use and public health by understanding how the public perceives the FDA’s JUUL ban policy.

## Methods

### Data Collection and Preprocessing

From June 22, 2022, to July 25, 2022, we collected 320,888 tweets related to e-cigarettes through the Twitter streaming application programming interface using a list of keywords such as e-cigarette, e-cig, and vaping [18,19]. To identify tweets related to the FDA’s proposed JUUL ban policy, we used further filtering by using lowercase matching with keywords such as “JUUL” and “ban.” We removed retweets containing the “RT @” keyword. We removed unrelated commercial and promotion tweets with the following keywords: “deal,” “supply,” “dealer,” “customer,” “discount,” “sale,” “free shipping,” “sell,” “$,” “%,” “dollar,” “offer,” “percent off,” “store,” “promo,” and “promotion.” After the initial filtering processes, we identified 63,286 tweets that might be related to the FDA’s JUUL ban policy.

### Feature Extraction of Twitter Users

#### Verify Status

To determine whether Twitter accounts with verified status have different attitudes from those not being verified, we included the verified status as one of the features for further analysis.

#### Geolocation of US Twitter Users

Twitter data that we collected contain the metadata about Twitter users including the location information. To compare Twitter users’ attitudes from various locations in the United States, we used population density data collected by the simplemaps database and categorized tweets into different geographic areas based on the top 2000 city names [20]. We used state names to identify the state-level tweets if Twitter users did not provide specific city information. Through city-state mapping, we combined these state-info-only tweets with the city-level corpus to form the state-level corpus. We used full names and abbreviations of states and cities for filtering. With the city-level corpus, we aimed to investigate whether Twitter users from rural or urban areas would exhibit different attitudes. Based on rural-urban differences in population density defined by the Degree of Urbanization level, we set urban areas where the population density is at least 1500 inhabitants per km^2^ and the rural regions where the population density is smaller than 1500 inhabitants per km^2^ [21]. We used Twitter users with state location information to compare the public attitude toward the JUUL ban policy across US states. Using the geolocation name as a filter, we obtained 17,007 relevant tweets from the United States.

#### Age, Gender, and Organization Estimation of US Twitter Users

We used the cutting-edge M3 model to predict Twitter users’ profile characteristics including age, gender, and whether they are affiliated with an organization, based on their profile images (preferred whenever available), screen names, and user profile descriptions [22]. The macro *F*_1_-scores of the M3 model for classifying gender, age, and organization were 0.918, 0.552, and 0.898 [22]. To enhance the accuracy of age prediction and categorize ages into meaningful groups, we divided users into 4 categories based on age: youth (13-18 years), young adults (19-29 years), middle-aged adults (30-39 years), and older adults (40 years or older). Gender was treated as a binary classification, with users classified as either male or female. The organization feature indicates whether the user is linked with an organization.

### Attitudes of Tweets Toward the JUUL Ban Policy

To avoid possible noise from the general discussion on JUUL and e-cigarettes and analyze Twitter user’s attitudes toward the JUUL ban policy more accurately, we used a deep learning algorithm coupled with human annotation to classify tweets into propolicy, antipolicy, neutral, and irrelevant categories. We first randomly selected 2600 tweets from the 17,007 relevant tweets. A total of 2 coders (PL and XL) used the induction method to code 600 tweets randomly chosen from the 2600 tweets independently to classify the tweets into propolicy, antipolicy, neutral, and irrelevant categories after coding the first 20 tweets together. The κ statistic on the 600 tweets was 0.87, indicating a strong agreement between the 2 coders. Any discrepancies were discussed within the group of 4 members (PL, XL, ZX, and DL) to achieve consensus. The 2 coders (PL and XL) continued to code the remaining 2000 tweets, each coding 1000 tweets independently. The manually coded 2600 tweets were used as the training data to train the deep learning algorithm to classify the remaining 14,407 JUUL ban policy–related tweets into propolicy, antipolicy, neutral, and irrelevant categories. We used the state-of-the-art deep learning transformer–based language model RoBERTa to label the attitudes of the other tweets [23]. This model was pretrained on the over 160 GB corpus. It achieved state-of-the-art performance on several language understanding tasks including question and answering, mutigenre language inference, and text entailment recognition [23]. Pretrained on a large corpus, it can generalize language understanding for various sequence classification tasks. We connected one layer of the feedforward neural network to the pretrained model to project the text embedding into predefined propolicy, antipolicy, neural, and irrelevant clusters. We randomly sampled 80% (2080/2600) of tweets as the training data and 20% (520/2600) as the validation to examine the model performance. The model indicated a solid ability to classify attitudes of tweets with a final *F*_1_-score of 0.850. Finally, we identified 2777 propolicy tweets, 2666 antipolicy tweets, 5037 neutral tweets, and 6527 irrelevant tweets.

### Logistic Regression on the Attitude Toward the JUUL Ban Policy

To examine the association of Twitter users’ characteristics with the Twitter user’s attitudes toward the JUUL ban policy, we applied a logistic regression model on Twitter users with either positive (propolicy) or negative (antipolicy) attitudes toward the JUUL ban policy. The binary outcome is the Twitter user’s attitude toward the JUUL ban policy, and the predictor variables include account verification status, geolocations, age, gender, and organization account or not. We used the 2-sample 2-tailed *t* test in the statistical analysis software R (R Core Team) for data analysis. The significance level of the test is 5%.

### Sentiment Analysis

We applied the VADER (Valence Aware Dictionary and Sentiment Reasoner) as the sentiment analyzer to measure the sentiment of each tweet. VADER is a widely used tool for sentiment analysis of social media data that can measure the sentiment polarity of text by computing a composite score ranging from –1 (extremely negative) to +1 (extremely positive), which has a precision of 0.99, recall of 0.94, *F*_1_-score of 0.96 [24].

### Topic Modeling Analysis

We used latent Dirichlet allocation (LDA) modeling to identify popular topics in those JUUL ban policy–related tweets within the propolicy and antipolicy groups. LDA is a generative text model that clusters words and terms in a given document and generates topics with keywords and corresponding weights indicating their likelihood of appearing [25]. To ensure consistency in the training model process, we converted all characters to lowercase and lemmatized all words using spaCy (Explosion AI). Additionally, we removed stop words, such as personal pronouns and prepositions, with the help of Natural Language Toolkit packages. We identified frequent bigrams (eg, JUUL ban) and trigrams (eg, food drug administration) using the *Gensim* package as single terms during model training to obtain precise and meaningful results. We selected the number of topics from 3 to 10 and determined the optimal number of topics using the coherence score of each LDA model result. Finally, we obtained the keywords of the fitted LDA topic model and the percentage distribution of each topic using the *pyLDAvis* package.

### Ethical Considerations

This is a secondary analysis of publicly available social media data. All the Twitter data have been deidentified before the data analysis. This study has been reviewed and approved by the Office for Human Subject Protection research subjects review board at the University of Rochester (study ID: STUDY00006570). Informed consent is waived due to secondary data analysis, and compensation is not needed for the secondary data analyses.

## Results

### The Longitudinal Trend of Public Attitudes Toward the JUUL Ban Policy on Twitter

**Figure 1:**
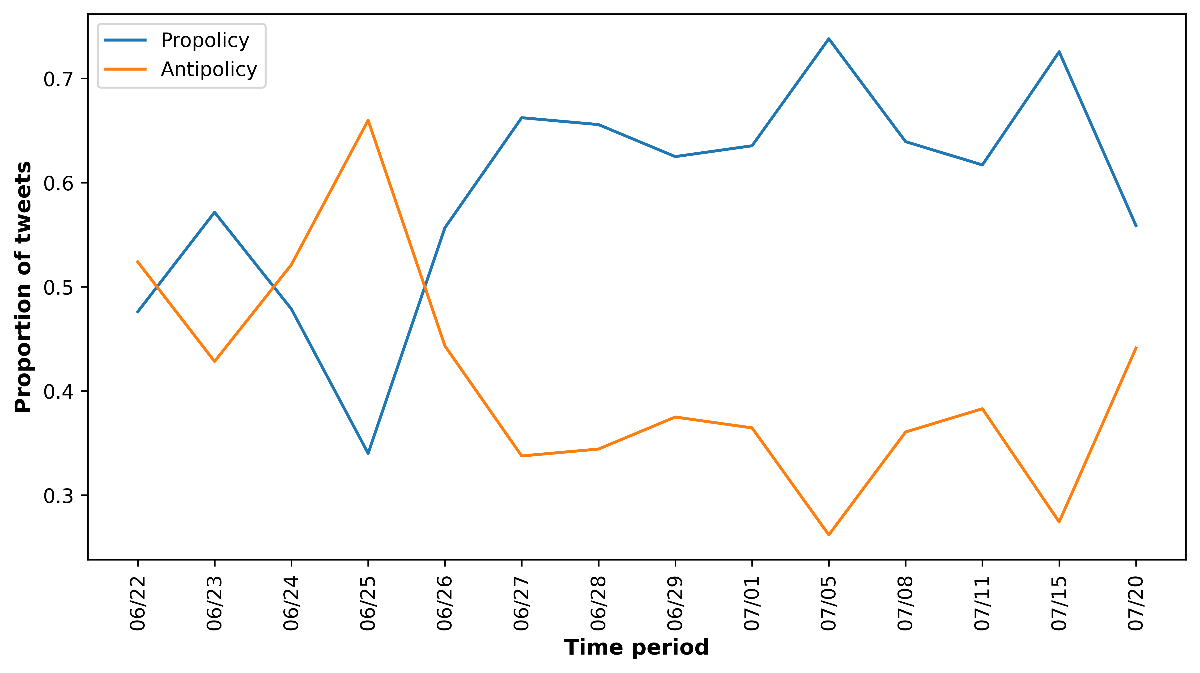
Public perceptions of the JUUL ban policy on Twitter over time. Each time interval is denoted by the start date.

To comprehensively capture all tweets relevant to the JUUL ban policy, including those before and after the official announcement, we collected tweets from June 22, 2022, to July 25, 2022. Using keywords related to the JUUL ban policy, we have identified 17,007 tweets from the United States that might be related to the JUUL ban policy. A total of 2 human coders double-coded randomly selected tweets about their relevance to the JUUL ban policy, reaching a κ statistic value of 0.87 (a strong interrater agreement). Furthermore, the trained state-of-the-art deep learning model (RoBERTa) with good model performance (*F*_1_-score=0.85) was used to determine the relevance of other tweets, resulting in 10,480 tweets in total relevant to the JUUL ban policy. Multimedia Appendix 1 shows an extreme surge in policy-related discussions on June 23, 2022, which declined rapidly within 5 days. Among 10,480 tweets related to the JUUL ban policy, 2777 (26.5%) tweets were propolicy, 2666 (25.44%) tweets were antipolicy, and 5037 (48.06%) tweets showed a neutral attitude toward the JUUL ban policy. This study focused on those tweets with apparent attitudes toward the JUUL ban policy, that is, propolicy and antipolicy tweets. Due to the small sample size, we combined adjacent days with fewer than 100 tweets per day into groups to ensure that the distribution of pro and antipolicy tweets is representative. Figure 1 shows the proportion of propolicy and antipolicy tweets over the study period. The proportion of antipolicy tweets reached the maximum on June 25, 2022, and gradually decreased afterward. The proportion of antipolicy tweets reached its lowest on July 5, 2022. In contrast, the proportion of propolicy tweets was relatively low at the beginning of the announcement of the JUUL ban policy. It increased afterward and stayed at a high level through the rest of the study period.

### Major Topics of Propolicy and Antipolicy Tweets Related to the JUUL Ban Policy

To understand possible reasons for either propolicy or antipolicy attitudes toward the JUUL ban policy on Twitter, we performed a topic modeling analysis on propolicy and antipolicy tweets, respectively. As shown in Table 1, there were 3 major topics in the propolicy tweets (n=2777), including “Against market surge of JUUL product” (n=1064, 38.3%), “JUUL causes youth addiction” (n=989, 35.6%), and “Negative health effect of JUUL” (n=724, 26.2%). The 3 major topics in the antipolicy tweets (n=2666) included “Ban cigarette instead of JUUL” (n=1176, 42.3%), “Emotional catharsis” (n=861, 31%), and “Against the irrational policy” (n=629, 22.7%). Based on the attitude classification by the RoBERTa model, we found the overall mean sentiment score of propolicy tweets is higher than antipolicy tweets. Based on the 2-sample 2-tailed *t* test, the propolicy tweets had a significantly higher mean sentiment score than antipolicy tweets, with statistical significance (*P*<.001). However, propolicy tweets do not always have a positive sentiment score, as shown in the average sentiment score from Table 1.

**Table 1:** Major topics discussed in propolicy and antipolicy tweets toward the JUUL ban policy.

Attitudes toward the JUUL ban policy and major topic		Tweets, n (%)	Description	Keywords	Average sentiment score	Example tweets
**Propolicy (n=2777)**						
	JUUL causes youth addiction	989 (35.6)	Twitter users complained that JUUL has contributed to an increase in vaping among youth and young adults.	ban, vape, cigarette, vaping, kid, product, tobacco, market, teen, people	–0.07	JUUL is guilty. A decade ago, they sent reps to high schools talking about the dangers of smoking, and claiming their products were safe to use, even for nonsmokers. They were actively recruiting teens.
	Against market surge of JUUL product	1064 (38.3)	Twitter users expressed a collective desire to resist the surging presence of JUUL in the market	market, cigarette, vaping, teen, company, ban, product, vape, pull, surge	0.075	“...you could say [JUUL] is a victim of its own success. You know, when it launched in 2015, JUUL hired young models and took out ads on Nickelodeon and Cartoon Network. These are channels kids watch.”
	Negative health effect of JUUL	724 (26.2)	The use of JUUL products has been linked to a higher incidence of lung cancer and cardiac ailments	product, vape, cigarette, health, ban, market, nicotine, public, vaping, teen	0.015	Thousands of todays WA State teens wont die from nicotine related cancer, lung, cardiac illness thanks to this long-awaited action. WA State still needs to act in 23: lower max nicotine level in all e-cigs, ban the youth attractive vape flavors, ban menthol cigarettes.
**Antipolicy (n=2666)**						
	Ban cigarette instead of JUUL	1176 (42.3)	The JUUL ban policy may drive the transition from vaping to cigarette smoking	ban, cigarette, vape, smoke, make, get, government, product, people, cig	–0.159	This would be an incredibly dumb move by the FDA, banning JUUL from the market yet letting all its competitors stay in? Why are they even considering this while cigarettes kill thousands each year!?
	Against the irrational policy	629 (22.7)	The JUUL ban policy is irrational considering other policies on gun and abortion	ban, get, abortion, gun, right, pod, go, buy, cigarette, vape	–0.191	Cool and normal country where you can carry a gun anywhere but cant buy a goddamn JUUL pod. Its great
	Emotional catharsis	861 (31)	Using strong language and emotional expressions to vehemently oppose the JUUL ban policy.	take, vape, go, away, right, f***, ban, get, smoke, cigarette	–0.166	JUUL has harmful chemicals? F***... I can’t believe it. F***... Brb, I need a smoke

**Figure 2:**
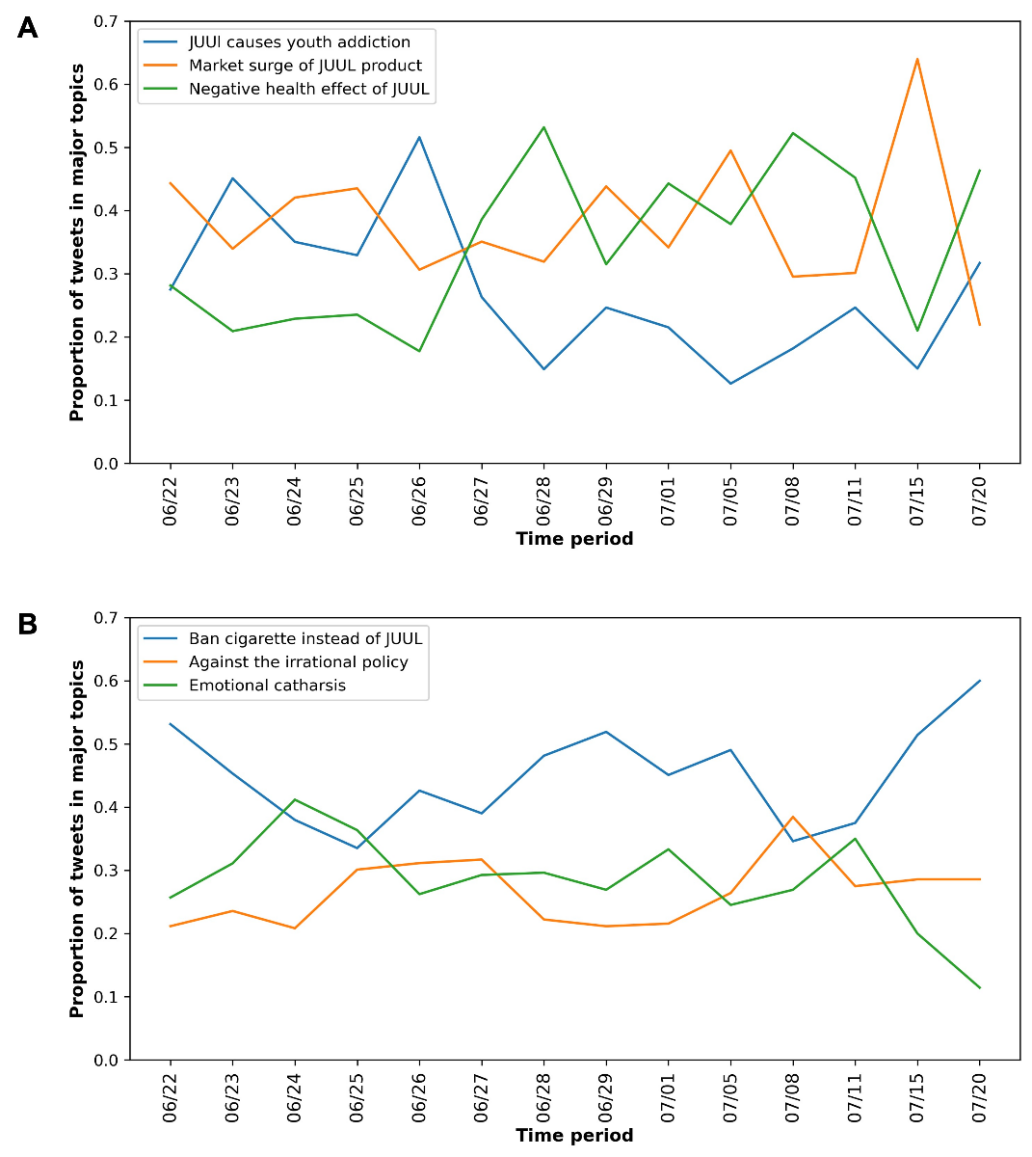
Major topics in propolicy and antipolicy tweets over time: (A) antipolicy tweets toward the JUUL ban policy and (B) propolicy tweets toward the JUUL ban policy. Each time interval is denoted by the start date.

Figure 2 shows the distribution of major topics from propolicy and antipolicy tweets over the study period. We noticed that in antipolicy tweets, the topic of “Ban cigarette instead of JUUL” dominated the antipolicy discussions most of the time in the study period. In contrast, no topic dominated the propolicy tweets during the study period. Among the propolicy topics, the topics of “Market surge of JUUL product” and “Negative health effect of JUUL” showed generally increasing trends, which may represent that as the JUUL ban was denied, people expressed more concern about the surge of JUUL products in the future market and more serious adverse effects of JUUL on the public health. In addition, “JUUL causes youth addiction” showed a decreasing trend opposite to the topic “Negative health effects of JUUL.” We noticed that the topic related to youth addiction contains an overall negative sentiment, for example, “You’re dead wrong. JUUL is harmful, especially for teens. Biden is using DPA on the baby formula.” Most of the tweets on this topic generally show a strong negative sentiment against the JUUL’s effect on kids.

### Associations of Twitter User Characteristics With Attitudes Toward the JUUL Ban Policy

**Figure 3:**
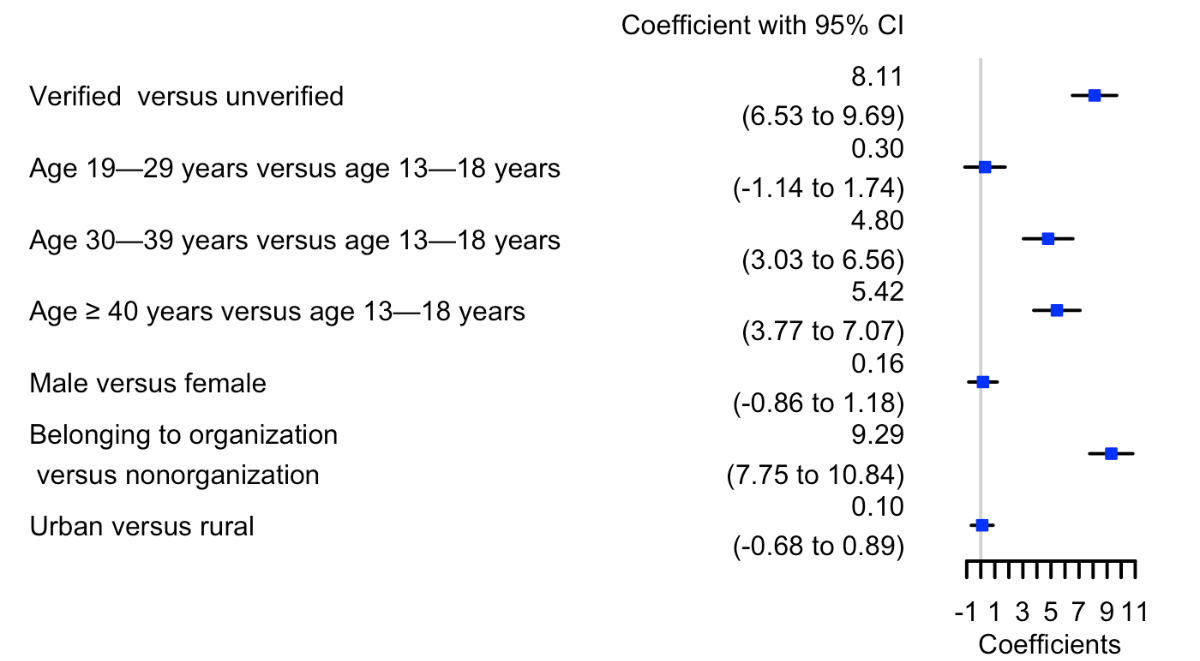
Comparison of the attitude toward the JUUL ban policy among different Twitter users. The estimated coefficient and their 95% CIs indicated a likelihood of positive attitudes toward the Food and Drug Administration JUUL ban policy.

A logistic regression model was used to examine the associations of Twitter user characteristics with the attitude toward the FDA JUUL ban policy. As shown in Figure 3, Twitter users with a verified status were significantly more likely to express a positive attitude toward the JUUL ban policy (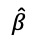
=8.11, 95% CI 6.53-9.69). Twitter users aged 30-39 years (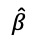 1=4.80, 95% CI 3.03-6.56) or 40 years or older (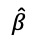 =5.42, 95% CI 3.77-7.07) were significantly more likely to show a positive attitude toward the FDA JUUL ban policy than those aged 13-18 years. In contrast, there was no significant difference in the attitude toward the JUUL ban policy between Twitter users in the 13-18 year age group and those in the 19-29 year age group (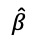
=.30, 95% CI –1.14 to 1.74). Twitter users who belong to an organization were significantly more likely to express a positive attitude toward the JUUL ban policy than those who do not belong to any organization (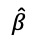
=9.29, 95% CI 7.75-10.84). No significant differences were observed between male and female Twitter users in their attitude toward the JUUL ban policy. In addition, the geolocation (urban vs rural) of Twitter users was not significantly associated with the Twitter users’ attitude toward the JUUL ban policy.

## Discussion

### Principal Findings

Using Twitter data from June 22, 2022, to July 25, 2022, this study examined the public perception and discussions of the FDA JUUL ban policy on Twitter. Overall, there were slightly more tweets with a positive attitude toward the FDA JUUL ban policy than those with a negative attitude. The propolicy tweets mainly focused on the potential harm of JUUL products, including youth addiction and adverse health effects, and the large market share of JUUL products. The antipolicy tweets focused on the complaints about the policy including banning cigarettes instead. Twitter users’ age, account status, and organization membership had significant associations with Twitter users’ attitudes toward the JUUL ban policy.

The longitudinal examination of the public attitude toward the FDA JUUL ban policy captured the dynamic changes in public attitudes and major events during the study period. Following the announcement of the FDA JUUL ban policy on June 23, 2022, there was a significant increase in antipolicy tweets, reaching a local maximum on June 25, 2022. The subsequent decreasing trend might be attributed to the federal appeals court’s temporary block of the government ban. During this period, the discussion of the market surge of JUUL products and their adverse health effects gradually emerged as the dominant propolicy topics, potentially reflecting dissatisfaction with the court’s decision on social media. However, on July 5, 2022, when the FDA stayed the MDO, we observed a sharp increase in the proportion of propolicy tweets, with over 50% focusing on discussing the market surge of JUUL products. These findings highlighted the importance of monitoring the public attitude over time and understanding the factors that might influence it, which can inform policy decision-making and public health interventions.

### Comparison With Prior Work

While the announcement of the JUUL ban policy in the United States might lead to a wide-range discussion on social media that is not limited to the United States, the responses to this ban might be different between the United States and other countries considering this policy only applied to the United States. To better understand how the public in the United States responds to this policy, we decided to focus on posts from the United States. In this study, we observed that there were similar proportions of propolicy and antipolicy tweets toward the JUUL ban policy from the United States. However, another study showed that antipolicy tweets were more prevalent than propolicy tweets regarding the JUUL ban policy [26]. However, while our study focused on the posts from the United States, that study analyzed the Twitter data not limited to the United States, which might lead to different results. In addition, our study has a much larger sample size than that study (n=10,480 vs n=2755).

Twitter users’ propolicy to the JUUL ban policy is primarily concerned with 3 major issues: youth addiction, the market surge of JUUL products, and their harmful health effects, which is consistent with US e-cigarette policies that respond to these major issues [1,27]. The high concentration of nicotine salt, sleek designs, and various flavors in JUUL products attracted many youth users and exponentially increased JUUL’s popularity and market share [28]. Many Twitter users were optimistic that the JUUL ban policy could help reduce JUUL use and protect the youth from the potential harm of JUUL products. Our sentimental analysis of these tweets revealed a pervasive pessimistic sentiment, with negative keywords such as “blame” and “harmful” appearing frequently (Multimedia Appendix 2). Specifically, “blame” appears 222 times in the topic related to youth addiction. For example, “JUUL is often blamed for the teenage vaping epidemic since its products contain high levels of nicotine, the same addictive chemical found in cigarettes.” Reflected by the negative sentiment score and large counting of the negative keywords, we identified a strong pessimistic feeling of Twitter users for the prevalence of JUUL among youth. This result was consistent with previous studies that negative attitudes toward e-cigarette use by youth were associated with stronger intentions to support policies aimed at reducing youth access to e-cigarettes [29-31].

Twitter users with an antipolicy attitude toward the JUUL ban policy tended to believe that conventional cigarettes, rather than JUUL products, should be banned. Some tweets suggested that individuals who use JUUL to quit smoking would switch back to cigarettes if JUUL products were banned. Other tweets compared the JUUL ban policy to the gun policy and abortion policy and considered none of them to be rational. The top negative polarized words and strong negative sentiment scores in these tweets suggest that many were emotional catharsis, often featuring rude language. Our findings suggest that restricting vaping (such as the JUUL ban policy) while leaving combustible cigarettes available could lead to antipolicy toward current e-cigarette regulatory policies (or support looser regulations on e-cigarettes). Therefore, a more comprehensive tobacco regulatory policy that targets cigarettes is warranted to mitigate the antipolicy sentiment for e-cigarette regulations [32,33]. Public health campaigns and interventions should focus on educating youth about the health effects of e-cigarette use and regulating the marketing and sale of these products to minors. Policy makers should consider the potential impact of e-cigarette policies on smokers who use e-cigarettes as a smoking cessation aid while prioritizing public health and reducing youth access to these products.

We conducted a logistic regression analysis to investigate features of Twitter users associated with the attitude toward the JUUL ban policy. Our findings indicated that verified Twitter users were more likely to support the JUUL ban policy. This result aligns with the work by Sirola et al [34] showing that verified Twitter users tend to engage in more positive interactions and have more followers than nonverified users. Additionally, we found that the older age group was more likely to support the JUUL ban policy than younger teenagers and adults. This propolicy attitude may reflect a negative perception of e-cigarettes among the older age group. A survey study on more than 13,000 young people aged between 15 and 34 years explored the prevalence and characteristics of JUUL products and questioned JUUL’s claims on targeting adult smokers and not marketing to youth [35]. A previous study showed that teenagers aged between 15 and 17 years had a 16 times greater chance of vaping than adults aged between 25 and 34 years [35]. Given the high prevalence of JUUL use in youth and young adults, it was not surprising to observe that they are more likely to be antipolicy toward the FDA JUUL ban policy than middle-aged or older adults. Our study did not observe a significant gender difference in the attitude toward the JUUL ban policy, consistent with the work by Bedi et al [36] showing no consistent gender differences in reasons for using e-cigarettes.

Geographically, we did not observe a significant difference in public perception of the JUUL ban policy between different states, as shown by the geolocational map of our study in Multimedia Appendix 3. This is not surprising given that the FDA ban on JULL is at the federal level and impacts all states. However, we can still observe which area in the United States showed relatively more support for the JUUL ban policy and which showed a more negative attitude. The states in the United States mainland with a relatively higher proportion of pro-JUUL ban policy tweets are South Dakota, Idaho, Oregon, Colorado, and Connecticut, where the proportions of tweets with a positive attitude are larger than 0.57. The states in the United States mainland with a relatively lower proportion of pro-JUUL ban policy tweets are Rhode Island and North Dakota, where the proportions are smaller than 0.39. The reasons underlying these differences need to be further investigated.

### Limitations

There were several limitations in this study. First, this study used a deep learning system to estimate the basic demographics of Twitter users, which might introduce some biases and inaccuracy since it makes demographic inferences based on the profile images (could be the images from others such as celebrities) or information provided by Twitter users. Twitter users’ demographics differ slightly from the US census data. Thus, the results from Twitter users may not represent the entire US population. Second, our current keyword list might not cover all relevant tweets to the JUUL ban policy, which might lead to some bias in the results. Third, we could not differentiate JUUL users from non-JUUL users. Therefore, we could not examine whether there is a difference in the attitudes toward the JUUL ban policy between JUUL users and non-JUUL users. Fourth, this study period may not reflect a longer trend of public attitudes toward the JUUL ban policy. Future studies may consider extending the study period for a more comprehensive understanding of this issue. Fifth, our use of VADER for sentiment analysis is rule based and sensitive to sarcasm, which might lead to some biased results. Sixth, considering Twitter’s strong contingent of antiregulatory voices that contribute disproportionately to tobacco control policy discourse, our Twitter data analysis might not fully represent the general public’s perceptions [37,38]. Additionally, since not all tweets contain valid location information of Twitter users, the data we collected only focuses on users willing to provide their geographic locations in the United States, which introduces a potential bias in our study. Therefore, considering the limitations mentioned above, our findings in this study may not be generalized to the whole population. Despite these limitations, it is pertinent to emphasize that our study’s focus on the US region remains relevant and significant, especially considering that the FDA’s JUUL ban policy was enacted within this jurisdiction. Hence, the data, albeit limited by the user’s willingness to share their location, still provides meaningful insight into the public perceptions of the policy in the United States, offering a valuable snapshot of the discourse among a segment of the population likely to be directly impacted by the regulation.

### Conclusion

In general, we found a mixed attitude toward the JUUL ban policy on Twitter during the study period from June 22, 2022, to July 25, 2022. The propolicy tweets mainly focus on the harm of JUUL products, while the antipolicy tweets mostly complain about the JUUL ban policy. Twitter users’ age, account verification status, and organization membership were significantly associated with their attitudes toward the JUUL ban policy. Our results have important implications for public health interventions and policy decision-making. Monitoring public perceptions and discussions about tobacco regulatory policies over time can inform policy decisions and provide insights into factors that might influence public perceptions and behaviors. This study highlights the role of Twitter users’ characteristics in their perceptions of the policy, which provided insights for policymakers who seek to understand how to target specific populations with messages and interventions to reduce the harm of tobacco product use on public health.

## Appendix

Multimedia Appendix 1 Number of tweets related to the JUUL ban policy over the study period. Each period is denoted by the start date of that period.

Multimedia Appendix 2 Top polarity words in propolicy and antipolicy tweets.

Multimedia Appendix 3 Proportion of propolicy tweets in different US states.

## Abbreviations

e-cigarette: electronic cigarette
FDA: Food and Drug Administration
LDA: latent Dirichlet allocation
MDO: marketing denial order
VADER: Valence Aware Dictionary and Sentiment Reasoner

## Data Availability

The data sets analyzed during this study are available from the corresponding author on reasonable request.
