# Supplementary material for: Public Perceptions and Discussions of the US Food and Drug Administration's JUUL Ban Policy on Twitter: Observational Study
Source: JMIR Form Res. 2024 Jul 11;8:e51327. doi: 10.2196/51327 (PMC11273066; doi:10.2196/51327)
Supplement: Multimedia Appendix 1 [file formative_v8i1e51327_app1.docx]

**
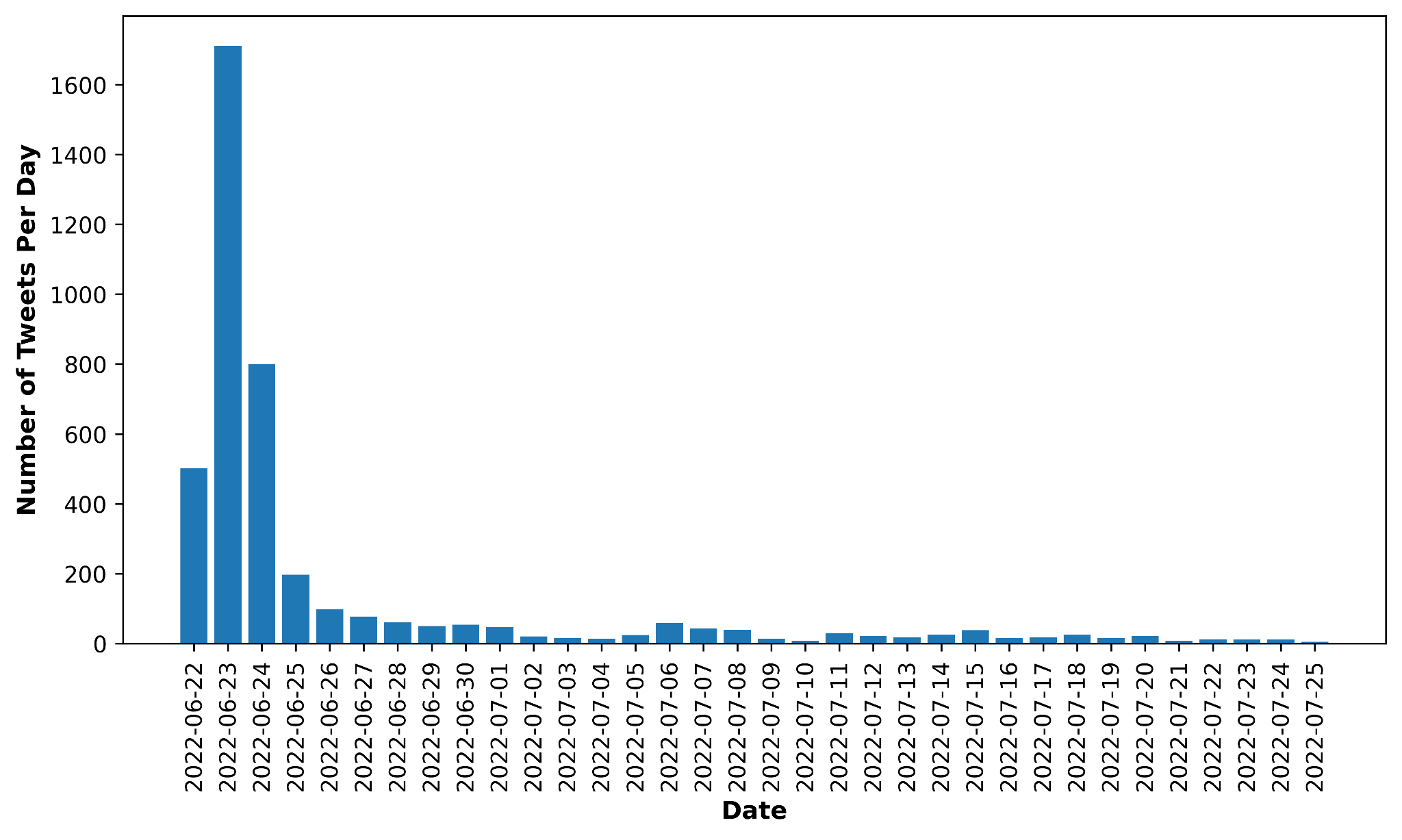
**

**Multimedia Appendix 1.** Number of Tweets related to the Juul ban policy over the study period. Each period is denoted by the start date of that period.
