# Supplementary material for: Public Perceptions and Discussions of the US Food and Drug Administration's JUUL Ban Policy on Twitter: Observational Study
Source: JMIR Form Res. 2024 Jul 11;8:e51327. doi: 10.2196/51327 (PMC11273066; doi:10.2196/51327)
Supplement: Multimedia Appendix 2 [file formative_v8i1e51327_app2.docx]

**Multimedia Appendix 2. Top polarity words in pro-policy and anti-policy tweets.**

| **Attitude toward the Juul ban policy** | **Top Positive Words** | **Top Negative Words** |
| --- | --- | --- |
| Pro-policy | Like (191)  Good (132)  Protect (69)  Want (66)  Thank (65)  Great (63)  Glad (53)  Help (52)  Optimistic (50)  Popular (49) | Blame (270)  No (148)  Anti (75)  Harm (74)  Dangerous (71)  Bad (69)  Stop (67)  Shit (41)  Problem (39)  Gun (38) |
|  |  |  |
| Anti-policy | Like (281)  Free (81)  Lol (63)  Good (56)  God (46)  Care (41)  Help (40)  Better (40)  Ok (40)  Joke (34) | No (245)  Fuck (132)  Gun (127)  Shit (105)  Bad (87)  Stop (64)  Stupid (63)  Wtf (57)  Kill (53)  Damn (51) |
|  |  |  |
