# Supplementary figures and images for: Public Perceptions and Discussions of the US Food and Drug Administration's JUUL Ban Policy on Twitter: Observational Study
Source: JMIR Form Res. 2024 Jul 11;8:e51327. doi: 10.2196/51327 (PMC11273066; doi:10.2196/51327)

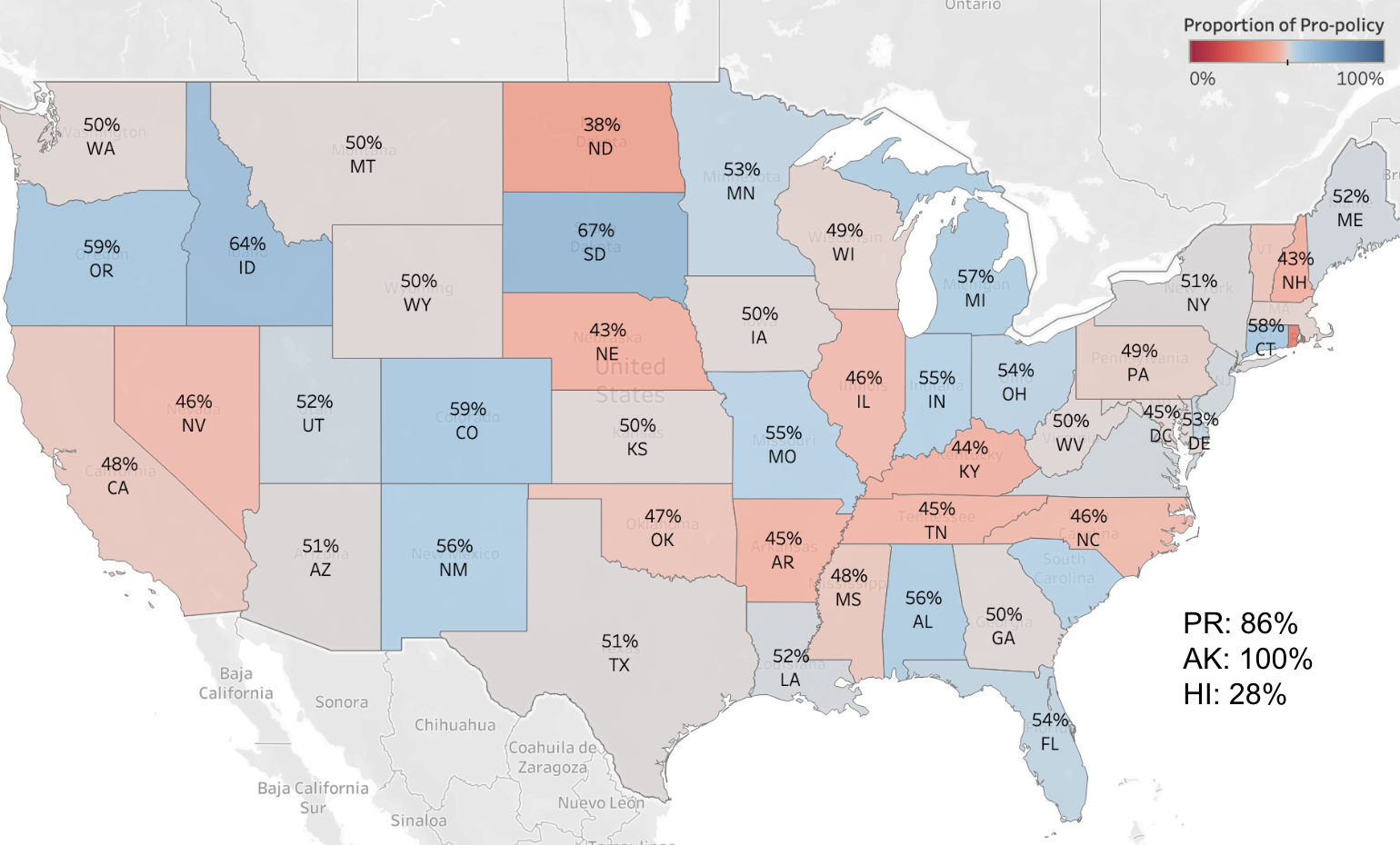


**Multimedia Appendix 3.** Proportion of pro-policy tweets in different US States.

Supplement: Multimedia Appendix 3 [file formative_v8i1e51327_app3.docx]
